# Mutational status of plasma exosomal KRAS predicts outcome in patients with metastatic colorectal cancer

**DOI:** 10.1038/s41598-021-01668-7

**Published:** 2021-11-22

**Authors:** Donatella Lucchetti, Ina Valeria Zurlo, Filomena Colella, Claudio Ricciardi-Tenore, Mariantonietta Di Salvatore, Giampaolo Tortora, Ruggero De Maria, Felice Giuliante, Alessandra Cassano, Michele Basso, Antonio Crucitti, Ilaria Laurenzana, Giulia Artemi, Alessandro Sgambato

**Affiliations:** 1 Department of Translational Medicine and Surgery, Università Cattolica del Sacro Cuore, 00168 Rome, Italy; 2 Department of Translational Medicine and Surgery, Fondazione Policlinico Universitario Agostino Gemelli IRCCS, 00168 Rome, Italy; 3 Division of General Surgery, Cristo Re Hospital, Rome, Italy; 4 Centro Di Riferimento Oncologico Della Basilicata (IRCCS-CROB), Rionero in Vulture, PZ Italy

**Keywords:** Cancer, Biomarkers, Oncology

## Abstract

Liquid biopsy has become a useful alternative in metastatic colorectal cancer (mCRC) patients when tissue biopsy of metastatic sites is not feasible. In this study we aimed to investigate the clinical utility of circulating exosomes DNA in the management of mCRC patients. Exosomes level and KRAS mutational status in exosomal DNA was assesed in 70 mCRC patients and 29 CRC primary tumor and were analysed at different disease steps evaluating serial blood samples (240 blood samples). There was a significant correlation between the extension of disease and exosomes level and the resection of primary localized tumor was correlated with a decrease of KRAS G12V/ D copies and fractional abundance in metastatic disease. CEA expression and liver metastasis correlated with a higher number of KRAS G12V/D copies/ml and a higher fractional abundance; in the subgroup of mCRC patients eligible for surgery, the size of tumor and the radiological response were related to exosomes level but only the size was related to the number of KRAS WT copies; both KRAS wild-type and mutated levels were identified as a prognostic factor related to OS. Finally, we found that 91% of mutated mCRC patients became wild type after the first line chemotherapy but this status reverted in mutated one at progression in 80% of cases. In a prospective cohort of mCRC patients, we show how longitudinal monitoring using exosome-based liquid biopsy provides clinical information relevant to therapeutic stratification.

## Introduction

Colorectal cancer (CRC) is the third most common type of cancer and the second most common cause of cancer deaths worldwide, not only due to the aggressive nature of the neoplasm but also for the frequent diagnosis in advanced stages^1,2^. In fact, about 50% of CRC patients are diagnosed at late stages, for which few treatment options are available and up to 50–70% of them develop distant liver metastases during follow-up^1,3^. Despite recent developments in clinical and translational research, numerous efforts are still needed in order to identify diagnostic, prognostic and predictive factors that could help clinicians in the choice of the right treatment, increase the benefit and the efficacy of therapy and improve the survival of patients. Nowadays, target therapy is one of the principal strategies for cancer treatment thanks to a well-developed knowledge of cancer biology and mutations^4^. In metastatic colorectal cancer (mCRC), RAS mutational status drives the clinicians in the choice of EGFR inhibitor therapeutic strategies^5^. Indeed, KRAS activating mutations result in the constitutive activation of downstream signaling pathways and confer resistance to cell surface receptor tyrosine kinases inhibitors as well as to EGFR-targeted monoclonal antibodies (anti-EGFR) (e.g., cetuximab and panitumumab). Therefore, assessing the KRAS mutational status of tumor cells has become an essential tool for managing patients with CRC. However, several studies have shown that distant metastases can have specific genetic alterations different from those in the primary tumor^6^. Thus, characterization of metastatic sites, besides primary tumor, is essential to correctly guide targeted therapies in mCRC patients. However, invasive biopsies of metastatic sites are not always feasible and repeated testing for real-time surveillance is often difficult. Liquid biopsy is a useful tool to overcome the above-mentioned problems allowing a non-invasive tumor molecular profiling by using tumor-derived biomarkers that can be isolated from body fluids of cancer patients, including peripheral blood.

Two of the most important sources of biomarkers in the field of liquid biopsy are cell-free DNA (cfDNA) and exosomes^7^. Currently, plasma-cfDNA is most used for genomic testing but exosomes, tiny bound sacs vesicles with size range of 30-200 nm, represent a suitable alternative for liquid biopsy and are increasingly establishing themselves as useful biomarkers^8–10^. Indeed, it has been shown that mutational analysis of plasma exosomal DNA (exoDNA) for BRAF, KRAS, and EGFR has high sensitivity compared to analysis of plasma cfDNA. Mouliere and colleagues showed that cfDNA is generally fragmented with fragment sizes being smaller in mutant (90–150 bp) compared to non-mutant cfDNA (250 to 320 bp)^11^. Kahlert and colleagues reported that double-stranded exoDNA is mostly bigger than cfDNA with a size range between 2.5–10 kB in pancreatic cancer patients^12^ thus suggesting that exoDNA is more suitable for mutational analyses. Indeed, in pancreatic cancer Allenson and colleagues reported a higher detection rate of KRAS mutations in exoDNA than in cfDNA by droplet digital PCR^7^. On the other hand, Bernard and colleagues reported no difference in KRAS mutation detection rate for localized and metastatic pancreatic cancer when profiling cfDNA or exosomal DNA^13^. However, they showed that the concordance of KRAS mutation detection in resected primary pancreatic tumors was greater for exoDNA than cfDNA.

To date, no studies have investigated the reliability and significance of Kras mutational analysis of exoDNA isolated from patients with mCRC. The purpose of the current study was to monitor the levels of plasma exosomes and their KRAS mutational status in mCRC patients from first line therapy to progression. In addition, we sought to determine whether plasma exosomal DNA KRAS mutational status correlated with clinical outcome and survival of mCRC patients.

## Material and methods

### Patients and methods

This is a monocentric prospective study approved by the Ethical Committee of the Catholic University School of Medicine (Rome, Italy) on July 30 2015 (PROT n. 17349/15). Two-hundred and forty patients with mCRC who underwent first line chemotherapy at Medical Oncology of Policlinico Universitario “A. Gemelli”-IRCSS between November 2015 to June 2019 were enrolled and 70 of them were included in this study.

Inclusion criteria were: (1) male or female subjects aged ≥ 18 years; (2) histologically proven metastatic colorectal adenocarcinoma; (3) evaluable or measurable disease at baseline; (4) first-line chemotherapy (CT) administration with or without target drugs (Bevacizumab, Cetuximab or Panitumumab) according to molecular characteristics; (5) known KRAS, NRAS and BRAF wild-type status of corresponding primary tumor or presence of KRAS G12V or G12D mutations assessed by immunohistochemistry; (6) no serious concomitant illnesses that could have affected treatment duration, short-time survival or the possibility of surgery. Exclusion criteria were: (1) previous malignant disease, besides CRC; (2) significant acute or chronic infections (HIV, HCV e HBV infections); (3) active autoimmune disease; (4) pregnancy or ongoing lactation; (5) any psychiatric condition that would prevent the understanding or rendering of informed consent; (6) NRAS or BRAF mutations and/or other KRAS mutations than G12V or G12D in primary tumor.

All patient’s data were collected anonymously; the study was conducted in accordance with the Declaration of Helsinki and consent for blood sampling and analyses was obtained by all patients according to the study protocol. Clinical characteristics of enrolled patients are reported in Supplementary Table 1.

Patients included in the study have been subjected to a first line chemotherapy (FOLFOX or FOLFIRI) associated or not with biological therapy (Bevacizumab, Cetuximab or Panitumumab depending on the molecular characteristics).

One blood sample (2 ml) was collected in EDTA from each enrolled patient according to the following timing scheme:

1. At the time of enrolment, before first chemotherapy administration (basal sample);
2. At each following radiological evaluation (every 2–3 months) (post-therapy);
3. At radiological progression, as assessed by computed tomography scan (progression);
4. In case of surgery: before surgery; at start of pre-operative chemotherapy (first line chemotherapy associated or not with biological therapy); after 4 and 30 days from surgery (post-4; post-30); during follow-up and at progression. Analyses of exosomal DNA were performed only in 10 patients among the 22 candidates for surgery because the remaining had Kras mutations different from G12V and G12D.

Plasma exosome levels were also assessed in twenty-nine patients with localized colon cancer to evaluate whether differences exist between metastatic and non-metastatic CRC patients.

Blood samples were centrifuged at 2500 rpm for 10 min and plasma samples were stored at − 80 °C before use.

### Exosome isolation and characterization

Exosomes were isolated from 1 ml of plasma by differential ultracentrifugation or using the Total Exosome Isolation KIT (Thermofisher, Massachusetts, US). In the first case, briefly, 1 ml of plasma collected was centrifuged at 3000×g for 30 min at 4 °C, to remove large debris. The supernatant was filtered using a 0.22-μm pore filter and centrifuged at 17,000×g for 30 min at 4 °C to remove microvesicles. Then, exosomes were pelleted at 120,000×g for 90’ at 4 °C. Exosome pellet was washed with 3 ml of 1 × PBS and pelleted again by centrifugation at 120,000×g for 90’ at 4 °C. The resulting pellet was either suspended in 100 µl of 1X PBS (0.1 µm filtered) for whole exosome assessments or further processed for DNA or protein extraction. The resulting exosome pellet was suspended in 100 µl of 0.1 filtered 1XPBS. The total exosomes level was expressed as µg/ml of initial plasma used to isolate exosomes after the resuspension in 100 µl of PBS.

Commercial kit was used according to manufacturer instructions.

Size and morphological analyses of isolated exosomes were carried out using dynamic light scattering and transmission electron microscopy, respectively, as previously described^14^.

### Droplet digital PCR

The droplet digitalPCR (ddPCR) was applied to DNA extracted by exosome (exoDNA) samples isolated from plasma to evaluate 37 WT KRAS patients and 33 mutant KRAS patients. ddPCR probes matching the G12V e G12D KRAS mutations were purchased from Bio-Rad. Droplet digital PCR allow to distinguish the mutation forms thanks to ddPCR probes (FAM probes for KRAS G12V or G12D gene mutation and HEX probe for WT gene) matching the G12V e G12D KRAS mutations and the WT gene in the same sample. We adopted the ddPCR KRAS G12/G13 Screening Kit to screen our KRAS WT patients (we could not know what mutation had occurred at progression step) for the following seven KRAS mutations in a single well: G12A, G12C, G12D, G12R, G12S, G12V, G13D.

ddPCR was carried out on a QX100ddPCR system (Bio-Rad Laboratories). A total volume of 22 µl PCR reaction mixtures was prepared with 10 µl of Supermix for probes without dUTP (Bio-Rad), 1 µl target primers/probe, and DNA sample/water (variable volume). The DNA template input volume used for the analysis was 10μL. Using the QX100 Droplet Generator (Bio-Rad) according to the manufacturer’s instructions, a mean of 14,000 droplets per sample were obtained from the PCR reaction. Samples were then transferred to a Bio-RadQX-100 droplet reader and analyzed based on fluorescence intensity by QuantaSoft v1.4.0.99 software from Bio-Rad. The DNA concentrations were estimated by the Poisson distribution. Fractional abundance (FA) was calculated as follows: FA (%) = [mutant copy/ (wild-type + mutant copy)] × 100**.**

### Statistical analysis

Overall survival (OS) was defined as the time from the date of systemic therapy initiation to the date of death or last follow-up. Progression free survival (PFS) was defined as the time from the date of systemic therapy initiation to the date the patient was taken off the treatment or last follow-up without recurrence. The Kaplan–Meier method was used to estimate OS and PFS, and a log-rank test was used to compare OS and PFS among patient subgroups.

Analysis of Variance (ANOVA) test was used to compare exosomes concentration and copies of KRAS among cases across time. Bonferroni correction for multiple testing was employed for post-hoc comparisons. The Mann–Whitney U test was applied to assess the association among the clinical characteristics, exosomes KRAS status and concentration. *P* values < 0.05 were considered statistically significant. Cut-off values were calculated as median of exosomes concentration or copies of KRAS. All statistical analyses were performed with the SPSS 23 (SPSS) software program.

### Ethics approval and consent to participate

The study was approved by the following institutional ethics review boards of Ethical Committee of the Catholic University Medicine (Rome, Italy) on July 30 2015 (PROT n. 17349/15). All study subjects provided written informed consent. The study was performed in accordance with the Declaration of Helsinki.

## Results

### Isolation and characterization of human plasma exosomes

The gold standard technique for isolation of human plasma exosomes is the differential ultracentrifugation, but it is difficult to translate into routine clinical diagnostics because is not suitable as a high throughput procedure. For this reason, we also tested a different commercially available method: Total Exosome Isolation Kit (Thermofisher, Massachusetts, US). Our data confirmed the superiority of differential ultracentrifugation to isolate the plasma exosomes with size range within 30 and 200 nm (Ultracentrifugation size peak = 160 nm) (Supplementary Fig. 1a, b, c) while the commercial kit, based on the precipitation technique, showed a size range that exceeded the 200 nm (295 nm) (Supplementary Fig. 1a, b, c). Moreover, the exosomal markers expression level was higher in exosomes isolated by ultracentrifugation compared to those isolated using the Thermofisher kit (Supplementary Fig. 1c, d). For this reason, despite its clinical limitations, we conducted the study purifying human plasma exosomes by differential centrifugation.

### Extraction of DNA from human plasma exosomes and its analysis by agilent bioanalyzer

Several studies report the use of Qiagen kits for isolation of exosomal DNA and so we decided to adopt them for our experiments^7,15^, but we did not verify whether there are kits that perform better than the kits proposed by Qiagen. Particularly, we tested two different kits for DNA isolation: the QIAamp1Circulating Nucleic Acid Kit (Qiagen, Santa Clarita, CA) and QIAamp MinElute Virus Spin Kit (Qiagen, Santa Clarita, CA). Our data confirmed that both methods allowed to obtain DNA from exosomes (exoDNA) with comparable size (~ 10 kb) but the concentration was higher using the QIAamp MinElute Virus Spin Kit compared to the QIAamp1Circulating Nucleic Acid Kit (respectively, 25 ± 6.2 ng/µl and 0.16 ± 0.04 ng/µl, respectively) (Supplementary Fig. 2). Therefore, exoDNA was isolated using the QIAamp MinElute Virus Spin Kit in our study. The goodness of the exoDNA was confirmed by its digestion with restriction enzymes and by Agilent Bionalyzer, as previously described^12^ (Supplementary Fig. 2).

### Characteristics of patients undergoing liquid biopsy and evaluation of exosomes concentration

**Figure 1:**
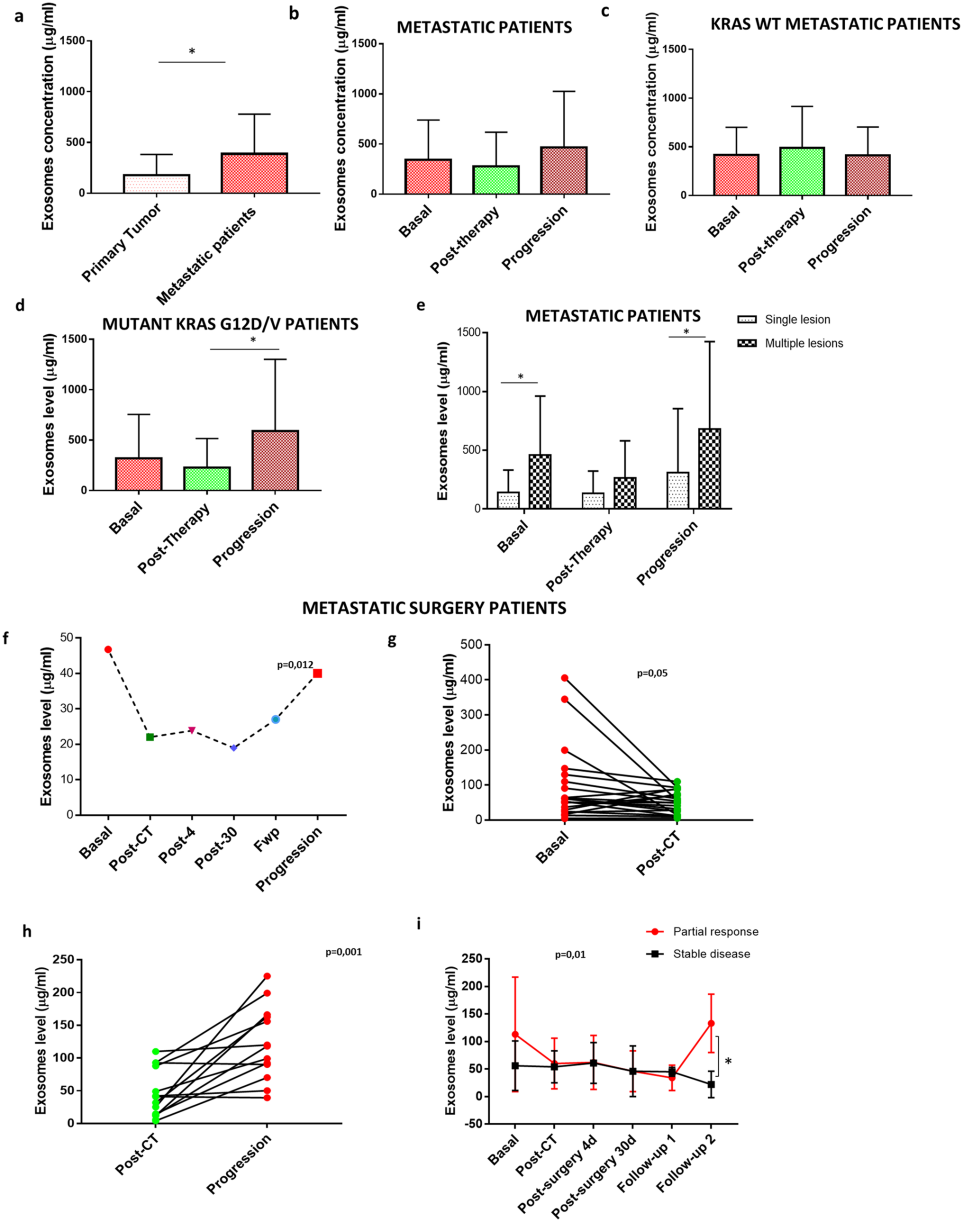
Evaluation of plasma exosomes levels correlated with clinical features of patients. (**a**) The graph show exosomes levels in patients with localized primary tumor (n = 29) compared to metastatic patients (n = 70); (**b**–**d**) evaluation of exosomes levels in three different subsets of patients: all metastatic patients (n = 70), *KRAS* wild type (n = 35) and *KRAS *^*G12D/V*^ mutated (n = 35); (**e**) the graph shows the correlation of exosomes levels of mutated patients with the presence of single (n = 6) or multiple lesions (n = 29) metastasis. (**f**) Trend of mean level of exosomes isolated from metastatic patients undergoing surgery (n = 22) at different time points from the day before start of therapy. (**g**,**h**) comparison among exosome concentration in metastatic patients undergoing surgery at basal, post-therapy and progression time point. (**i**) stratification of exosomes level in CRC metastatic patients candidate to surgery according to the radiological response (n = 5 for stable response; n = 17 for partial response).

Study overview and patient stratification are presented in Supplementary Fig. 3. A total of 240 blood samples from 70 patients with metastatic disease (mCRC) and 29 blood samples from patients with localized resectable CRC primary tumors were analysed for exosomes level. Among 70 metastatic patients, 22 were candidates for liver metastasis resection surgery after the first line of chemotherapy. Exosomes concentration was defined as μg per mL of plasma. Localized pre-surgical CRC patients displayed lower levels of exosomes (179.9 ± 37.5 μg/mL) compared to mCRC patients (388.6 ± 57.67 μg/mL) (*p* = 0.0098) (Fig. 1a). Exosomes levels decreased in mCRC patients after therapy and increased during disease progression although differences did not reach statistical significance (Fig. 1b). Exosomes levels were non significantly different between *KRAS *^*G12D/V*^ vs *KRAS WT* metastatic patients before therapy (basal step 320.0 ± 430 vs 337.3 ± 283 *p* = 0.85) (Fig. 1b–d). Stratifying patients based on the presence or absence of *KRAS*^*G12D/V*^, we found that the *KRAS wild type* cohort did not show variations in exosomes levels among the analyzed steps (Fig. 1c) while mutated patients showed a significant increase of exosomes levels at progression (post-therapy *KRAS*^*G12D/V*^ vs progression disease (PD) *KRAS *^*G12D/V*^* p* = 0.026) (Fig. 1d). Moreover, the exosomes level at progression was significantly higher in mutated patients compared to wild type counterpart (PD *KRAS*^*G12D/V*^ vs PD wild type *p* = 0.0019). In mCRC patients, plasma exosomes level was always higher in patients with multiple lesions compared to patients with single metastasis and the difference reached significance at basal and progression steps (basal single lesion vs basal multiple lesions *p* = 0.046; progression single lesion vs progression multiple lesions *p* = 0.037) (Fig. 1e, Table 1). Moreover, exosomes plasma levels changed in mCRC patients candidates to surgery and correlated with each treatment step, resulting significantly reduced after chemotherapy and surgery, compared to basal level, and significantly increased at progression (Fig. 1f–h). When mCRC patients candidate to surgery were stratified according to the radiological response to treatment, those with a stable disease (SD) showed a lower concentration of exosomes than those who responded partially (RP) to therapy at second follow up step (RP vs SD: *p* = 0.026) (Fig. 1i) (Table 2). Plasma exosomes levels were not correlated with progression free nor with overall survival in mCRC patients (data not shown).

**Table 1:** Correlation between plasma exosomes level, KRAS copies and clinical-pathological characteristics in G12D/V metastatic patients.

Copies KRAS G12D/V	Mutated patients		*P-value*
	Resection	Not resection	
Basal	50.5 ± 80.0	88.8 ± 77.3	**0.03**
Post-therapy	4.3 ± 9.1	3.2 ± 10.1	0.796
Progression	45.1 ± 46.1	77.3 ± 86.5	**0.029**

Fractional abundance (%)	Mutated Patients		*P-value*
	Resection	Not resection	
Basal	12.2 ± 14.6	28.9 ± 15.6	**0.021**
Post-therapy	2.4 ± 5.7	0	–
Progression	13.2 ± 10.9	27.2 ± 18.7	0.067

Exosomes level (µg/ml)	Number of lesions		*P-value*
	Single lesion	Multiple lesions	
Basal	142.3 ± 190.4	382.7 ± 479.4	**0.046**
Post-therapy	133.6 ± 189.9	266.4 ± 314.9	0.326
Progression	309.1 ± 545.5	680.0 ± 744.5	**0.037**

Copies KRAS G12/V	CEA		*P-value*
	Negative	Positive	
Basal	25.6 ± 21.5	104.4 ± 95.0	**0.003**
Post-surgery	6.1 ± 12.3	1.8 ± 6.0	0.649
Progression	24.6 ± 28.0	85.0 ± 76.4	**0.013**

Fractional abundance (%)	CEA		*P-value*
	Negative	Positive	
Basal	10.6 ± 14.2	24.0 ± 16.6	0.059
Post-surgery	2.3 ± 6.4	0.74 ± 2.4	0.904
Progression	9.9 ± 12.6	25.3 ± 15.4	**0.028**

### Liquid biopsy is suitable for detection of wild type and mutants KRAS in exoDNA by digital PCR

**Figure 2:**
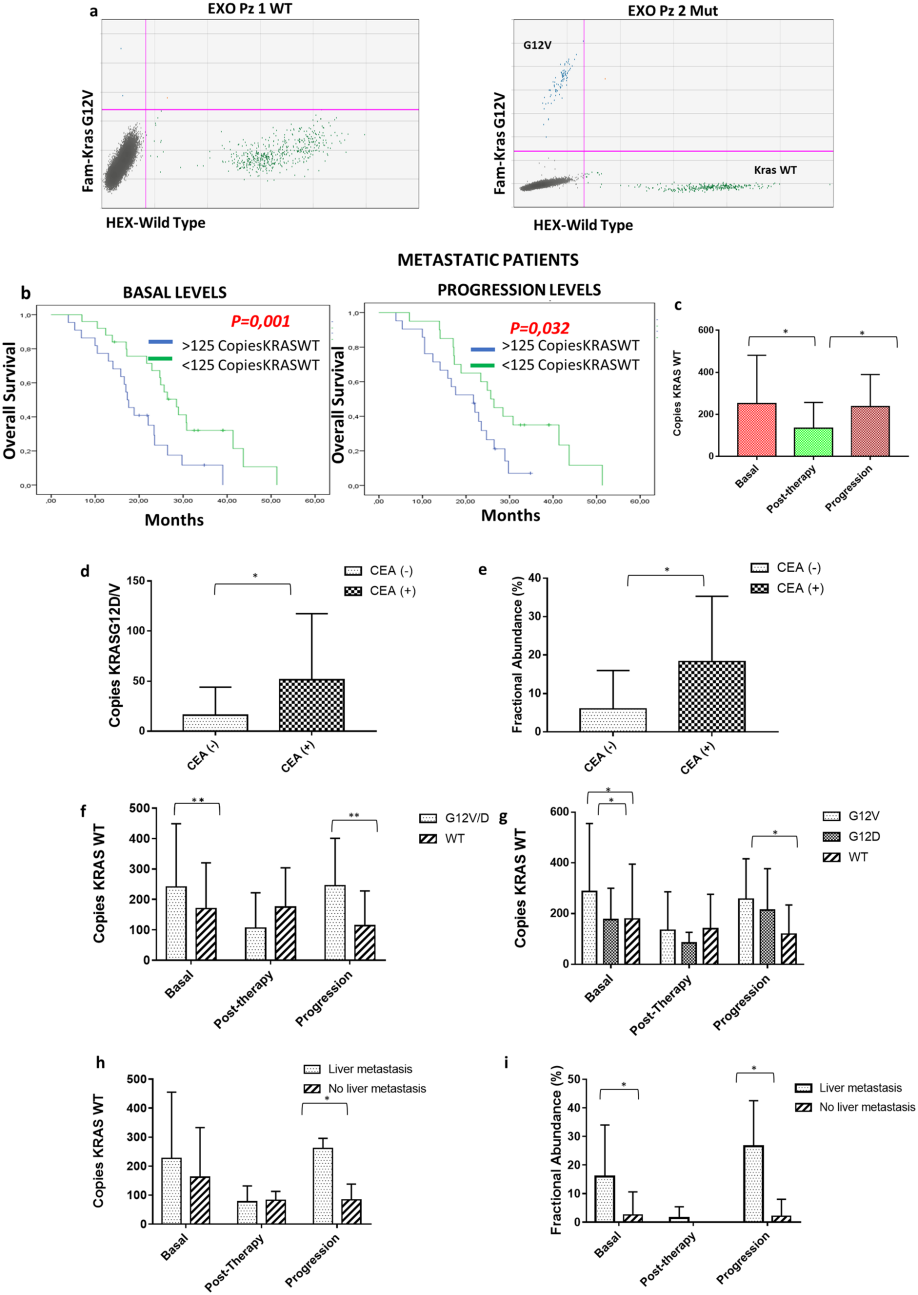
KRAS detection in plasma exosomes. (**a**) Representative 2D intensity scatter plot of wild-type and mutant amplicon for *KRAS*^*G*12*V*^ in two mCRC patients (wild-type and *KRAS*^*G12V*^ mutated); grey, no DNA; blue, mutant; green, wild type; (**b**) patients with a low *KRAS* WT amplicon (< 125 *KRAS* WT) displayed a significantly longer median OS duration than patients with a high (> 125 *KRAS* WT amplicon) *KRAS* WT amplicon; (**c**) number of *KRAS* WT and *KRAS*^*G12D/V*^ amplicon in mCRC patients at different time points (basal, post-therapy, progression); (**d**,**e**) amount of KRAS^*G12D/V*^ and the value of fractional abundance of mutated KRAS at progression in patients positive for CEA compared to patients negative for this marker; (**f**,**g**) amount of KRAS WT copies and the value of fractional abundance according to KRAS status; (**h**, **i**) amount of KRAS WT and the value of fractional abundance according to liver metastases. FAM = probe that recognize KRAS mutations; HEX = probe that recognize KRAS wild type.

*KRAS*^*G12D/V*^ mutant and wild-type amplicon were analyzed by droplet digital PCR (ddPCR) in plasma exosomes of 60 mCRC patients enrolled in this study (Fig. 2a). The wild type cohort of patients was analyzed by *KRAS*^*G12/13*^ screening kit. To confirm the correct identification of *KRAS* mutant and wild-type amplicon we used three colorectal cancer stem cell lines (CSC1 carrying mutated *KRAS*^*G12V*^, CSC2 carrying *WT KRAS* and CSC3 carrying mutated *KRAS*^*G12D*^) (Supplementary Fig. 4). Moreover, we also searched for *mutant* and *wild type KRAS* in exosomes released by the same cancer cell lines (Supplementary Fig. 4).

In 7 out of 60 patients (11.6% of total) the *KRAS* mutational profile was different from the primary CRC tumor tissue. A cutoff value of 125 copies/ml of *WT KRAS* gene (Fig. 2b) allowed to discriminate between mCRC patients with significantly different overall survival both at basal and progression time (*p* = 0.001 and *p* = 0.032 respectively) but no correlation was observed with progression free survival (data not shown). *KRAS WT* amplicon post-therapy was significantly lower compared to both basal and progression levels (*p* = 0.04) (Fig. 2c). Stratifying patients based on the expression of CEA, we found that the copies of *KRAS *^*G12/V*^ and the fractional abundance of mutated KRAS at progression was greater in CEA positive mCRC patients compared to negative ones (*KRAS *^*G12/V*^ progression, *p* = 0.024; FA progression, p = 0.022) (Fig. 2d,e and Table 3). *KRAS*^*WT*^ copies were significantly higher in the mutated groups compared to the *wild type* one both at basal and progression steps (*p* = 0.011; 0.001) (Fig. 2f, g; Table 3). Moreover, mCRC patients with liver metastases showed an increase of *KRAS *^*G12D/V*^ copies at basal time point and of fractional abundance at both basal and progression steps (*KRAS *^*G12D/V*^ basal, *p* = 0.015; FA basal, *p* = 0.020; FA progression*, p* = 0.030) (Fig. 2h, i; Table 3).

**Table 2:** Correlation between plasma exosomes level, KRAS copies and clinical-pathological characteristics in metastatic patients undergoing surgery.

Fractional abundance (%)	CEA (Surgical treated patients)		*P-value*
	Low	High	
Basal	10.82 ± 17.13	36.0 ± 22.1	**0.002**
Post-surgery	0	0	–
Progression	2.58 ± 5.17	27.81 ± 16.61	**0.002**

Exosomes level (µg)	Radiological response (Surgical treated patients)		*P-value*
	RP	SD	
Basal	104.2 ± 113.86	56.1 ± 45.26	0.359
Post-CT	60.1 ± 46.9	54.0 ± 29.22	1.000
Post-surgery (4dd)	62.8 ± 49.2	61.6 ± 37.9	0.905
Post-surgery (30 dd)	45.9 ± 37.8	46.5 ± 46.0	1.000
Follow-up	34.7 ± 23.3	45 ± 8.0	0.548
Progression	133.7 ± 53.46	21.6 ± 24.5	**0.026**

Copies KRAS WT	Tumor size (Surgical treated patients)		*P-value*
	< 3 cm	> 3 cm	
Basal	100.2 ± 48.5	328 ± 202.4	**0.032**
Post-CT	156 ± 180.5	88.0 ± 129.12	1.000
Post-surgery (4dd)	188.0 ± 212.4	154 ± 105.67	1.000
Progression	227.0 ± 61.9	250.6 ± 197.2	0.610

Exosomes level (µg/ml)	Tumor size (Surgical treated patients)		*P-value*
	< 3 cm	> 3 cm	
Basal	93.0 ± 113.7	93.5 ± 97.5	0.699
Post-CT	81.0 ± 45.7	36.4 ± 26.3	**0.013**
Post-surgery (4dd)	74.4 ± 48.0	49.5 ± 41.9	0.197
Post-surgery (30 dd)	38.1 ± 29.4	53.1 ± 45.7	0.799
Follow-up	36.6 ± 18.2	40.07 ± 23.9	0.730
Progression	123.9 ± 61.8	107.7 ± 72.9	0.945

**Table 3:** Correlation between exosomes level, KRAS copies and clinical-pathological characteristics in metastatic CRC patients.

Level (µg/ml)	Number of lesions		*P-value*	
	Single lesion	Multiple lesions		
Basal	142.3 ± 190.4	382.7 ± 479.4	**0.046**	
Post-therapy	133.6 ± 189.9	266.4 ± 314.9	0.99	
Progression	309.1 ± 545.0	680.0 ± 744.0	**0.037**	

KRAS WT copies	KRAS mutational status			*P-value*
	KRAS G12V	KRAS G12D	WT	
Basal	287.3 ± 268.9*	176.0 ± 124.7*	179.3 ± 216.4*	**0.001**
Post-therapy	134.2 ± 152.7	84.4 ± 41.9	140.0 ± 136.7	0.749
Progression	257.8 ± 159.05*	214.6 ± 163.8	119.8 ± 115.6*	**0.003**

KRAS WT copies	KRAS mutational status		*p-value*	
	KRAS G12V	WT		
Basal	241.3 ± 208.7	170.3 ± 150.0	**0.011**	
Post-therapy	175.0 ± 129.5	106.7 ± 116.3	0.645	
Progression	245.3 ± 156.0	114.0 ± 114.6	**0.001**	

KRAS WT copies	Liver metastases		*p-value*	
	Yes	No		
Basal	227.1 ± 228.6	170.3 ± 150.0	0.146	
Post-therapy	175.0 ± 129.5	106.7 ± 116.3	0.803	
Progression	245.3 ± 156.0	114.0 ± 114.6	**0.009**	

KRAS G12D/V copies	Liver metastases		*p-value*	
	Yes	No		
Basal	51.04 ± 109.3	6.0 ± 11.9	**0.015**	
Post-therapy	2.1 ± 6.6	8.0 ± 16.0	0.627	
Progression	43.6 ± 60.8	8.6 ± 17.7	0.067	

Fractional abundance (%)	Liver metastases		*p-value*	
	Yes	No		
Basal	15.7 ± 18.3	2.5 ± 8.1	**0.020**	
Post-therapy	1.38 ± 4.5	0 ± 0	0.787	
Progression	16.4 ± 16.8	2.1 ± 6.1	**0.030**	

KRAS G12/V copies	CEA		*P-value*	
	Low	High		
Basal	17.1 ± 24.53	59.6 ± 126.3	0.163	
Post-surgery	5.0 ± 11.3	1.53 ± 5.5	0.649	
Progression	16.1 ± 28.0	51.4 ± 66.9	**0.024**	

Fractional abundance (%)	CEA		*P-value*	
	Low	High		
Basal	6.4 ± 11.3	17.3 ± 19.2	0.077	
Post-surgery	2.01 ± 6.0	0.62 ± 2.2	0.896	
Progression	5.8 ± 10.3	18.3 ± 17.7	**0.022**	

**Figure 3:**
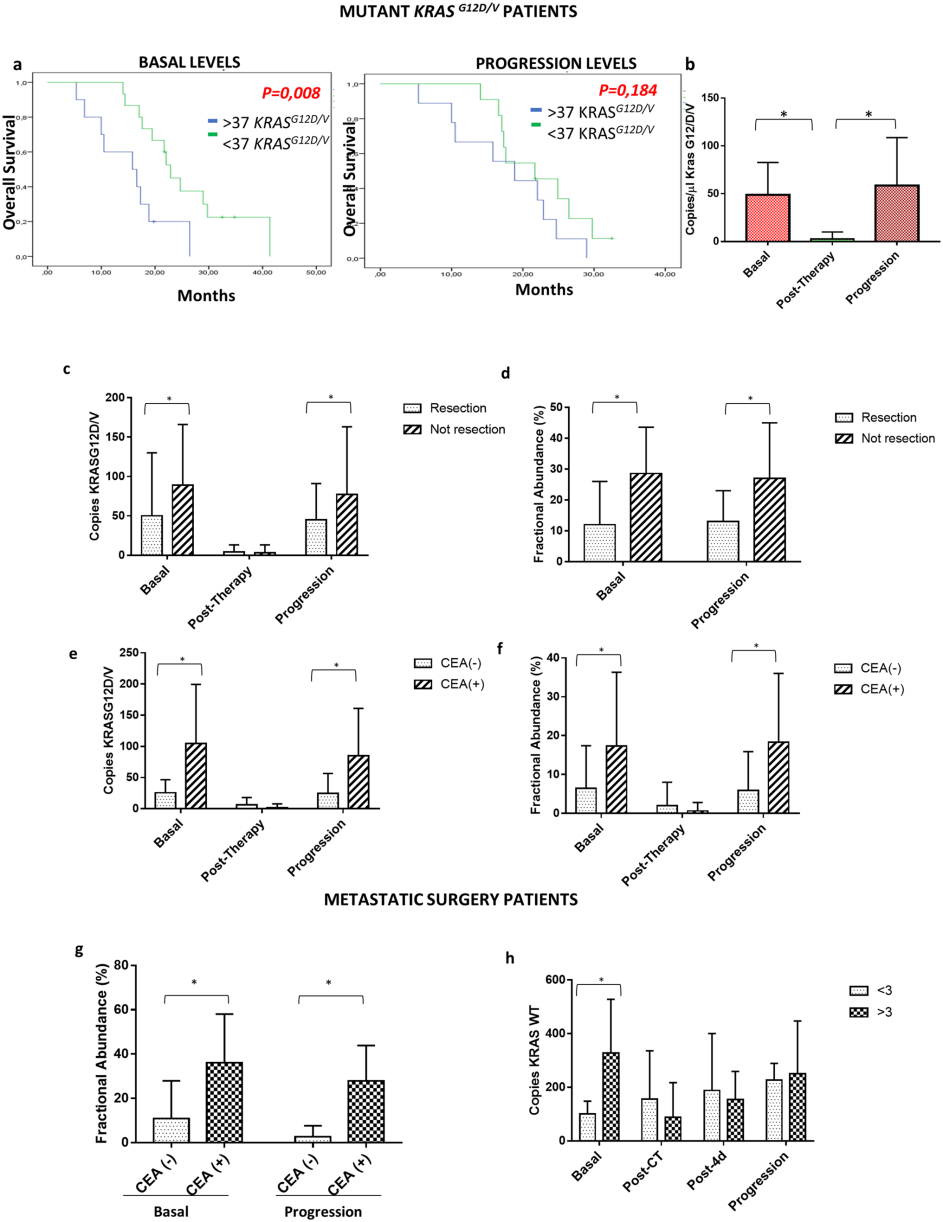
Correlation between KRAS mutational status and clinical features of patients. (**a**) Patients with a low *KRAS*^*G12D/V*^ amplicon (< 37 *KRAS* WT) had a significantly longer median OS duration than patients with a high (> 37 *KRAS* WT amplicon) *KRAS* WT amplicon; (**b**) copies of *KRAS*^*G12D/V*^ decreased after therapy and increased at progression time; (**c**,**d**) The patients not undergoing to resection surgery of the primary tumour have a number of *KRAS*^*G12D/V*^ copies higher compared to those who have undergone surgery; it’s the same for the fractional abundance parameter; (**e**,**f**) patients with positive expression of CEA have a number of KRAS^*G12V/D*^ copies and a fractional abundance higher than CEA negative patients; (**g**) fractional abundance in metastatic surgery patients according to CEA expression at basal and progression time point; (**h**) amount of KRAS WT according to size of tumor in metastatic surgery patients.

A cutoff value of 37 copies/ml of mutated *KRAS *^*G12/V*^ was able to separate patients with different overall survival at basal time (*p* = 0.008) but not at progression of disease (*p* = 0.184) (Fig. 3a). The number of KRAS mutated copies decreased significantly post-therapy (after first line of chemotherapy) compared to basal time and subsequently increased at progression (*p* = 0.0047) (Fig. 3b). Our liquid biopsy analysis showed that in the mutated cohort the *KRAS *^*G12/V*^ copies/ml as well as the fractional abundance of mutated KRAS were lower in patients undergoing resection surgery of the primitive tumor compared to patients that were not eligible for resection surgery (*KRAS *^*G12/V*^ basal, *p* = 0.03; *KRAS *^*G12/V*^ progression, *p* = 0.029; FA basal, *p* = 0.021) (Fig. 3c, d, Table 2) and were higher in CEA positive (> 5 ng/ml) compared to CEA negative (< 5 ng/ml) patients (*KRAS *^*G12/V*^ basal, *p* = *0,003*; *KRAS *^*G12/V*^ progression, *p* = 0.029; FA progression = *p* = 0.028) (Fig. 3e, f, Table 2). In mCRC patients undergoing liver surgery the stratification based on CEA expression allowed to identify a correlation between fractional abundance and *KRAS* mutation at basal and progression time-point (*p* = 0.002; *p* = 0.002) (Fig. 3g, Table 2). Moreover, we showed that the number of KRAS^*WT*^ copies correlated with the size of the tumor in mCRC patients undergoing surgery at basal time point (cut-off 3 cm; *p* = 0.032) (Fig. 3h and Table 2).

### Appearance and disappearance of RAS mutant clones in exosomal plasma during tumor progression

Cancers evolve by a reiterative process of genetic diversification and clonal selection. Genomic studies performed through liquid biopsies in mCRC patients have demonstrated that RAS mutant clones might appear during pressure of therapy, generating the needed of mutations-guided adaptive therapeutic strategies^16^. Similarly, the disappearance of RAS mutant clones in plasma has been more recently reported, supporting the perturbation of mutational status during the progression of tumor disease^17^. In our KRAS mutated cohort of mCRC patients, 12% of patients did not show the same KRAS mutation at progression disease step (Supplementary Fig. 5), three patients identified as *KRAS WT* by tissue biopsy of primary cancer were found to be mutated by liquid biopsy and 4 *KRAS mutated* patients were not mutated on exosomal DNA at disease progression. In addition, in 10 patients, at basal and at the time of progression, KRAS mutational status was discordant compared to baseline (5 KRAS ^*G12V/D*^ mutated patients at basal did not show mutated clones at progression while mutated clones were identified at the time of disease progression in 5 KRAS^*WT*^ patients at basal). Moreover, the 90% of mCRC mutated cohort patients after the first line chemotherapy became wild type but, in 85% of cases this status reverted in mutated one at progression (Table 4).

**Table 4:** 90% of mutated mCRC patients became wild type after the first line chemotherapy but this status reverted in mutated one at progression in 85% of cases.

Mutated patients	Basal (G12D/V copies)	Post-therapy (G12D/V copies)	Progression (G12D/V copies)
1	44	0	60
2	48	0	62
3	42	0	43
4	28	0	121
5	73	0	0
6	90	0	0
7	63	0	62
8	20	0	96
9	34	23	59
10	20	0	48
11	33	0	168
12	71	0	0
13	86	24	32
14	32	0	30
15	72	0	0
16	40	0	90
17	22	0	30
18	24	0	138
19	24	0	60
20	131	0	38

These data confirm the real need to monitor mCRC mutational status evolution at each step from the start of cancer therapy in order to be able to identify the most appropriate therapy.

## Discussion

Several evidences have suggested that tumor progression and treatment can select sub-clones of cancer cells that compete by strong selective external pressure^18^. Currently, the molecular characteristics of solid tumors are established by the resection of tumoral tissue after surgery or by tissue biopsy. Tissue samples for molecular characterization have many disadvantages: (1) do not represent the entire lesion due to tumor heterogeneity; (2) the samples cannot be repeated over time during the therapy and monitored until disease progression. This pitfalls can be overcome by liquid biopsy which requires a simple blood sampling to replace the use of tumor tissue in a non-invasive way and to allow the genetic characteristics of a malignant tumor by analysing tumor-derived components released into the circulation^19^.

Liquid biopsy might represent a dynamic tool suitable to catch both cancer heterogeneity and clonal evolution over time. Some evidence provided that the analysis of circulating tumor DNA (ctDNA) in blood samples could be an appropriate surrogate of tumor biopsy for the detection of RAS mutations, monitoring the temporal heterogeneity of a cancer during targeted therapies^17,20^. The analysis of cells and / or circulating DNA, mRNA and microRNA, however, has highlighted a series of technical issues that, so far, have significantly limited the clinical impact of this technique^14^. Cancer cells release small vesicles called exosomes into the circulation, which carry key elements such as DNA, microRNA and proteins that can became potential biomarkers for cancer disease. Exosomes have emerged as key mediators of cell-to-cell communication within the tumor microenvironment and cancer metastasis. Indeed, several studies have shown that cell-to-cell communication via extracellular vesicles, mainly exosomes, between primary tumor cells and the microenvironment of distant organs (local stroma and immune cells) is crucial for pre-metastatic niche formation and metastasis, favouring survival and grow of metastatic cells in a very hostile microenvironment^21^. Several studies show that circulating exosomes in plasma are increased in cancer patients compared to health controls^22,23^. It has been suggested that the level of exosomes in body fluids can serve as a potential diagnostic and/or prognostic biomarker in cancer patients and several studies show that cancer development can be monitored by analysing the level of exosomes in biofluid samples^22–25^. The features of exosomes make them ideal candidates for liquid biopsy-based biomarkers. In fact: (i) exosomes are tissue-specific; (ii) cargo of exosomes (RNA and DNA) is protected from nuclease and so the nucleic acids material is more suitable for molecular analyses than plasma cell-free DNA. Our study confirm the usefulness of exosome-based liquid biopsy as a tool to evaluate mCRC disease characteristics at baseline (before therapy) and its evolution during chemotherapy, showing that: (1) there was a significant correlation between the extension of disease (multiple versus single lesion) and exosomes level; (2) despite similar baseline exosomes levels in KRAS WT and mutated patients, the latter showed more changes according to treatment steps with a significant increase at progression; (3) the resection of primary tumor was correlated with a decrease of KRAS ^*G12V/D*^ copies and fractional abundance; (4) CEA expression correlated with a higher number of mutated KRAS copies/ml and a higher fractional abundance; (5) in the subgroup of mCRC patients eligible for surgery, the size of tumor and the radiological response were related to exosomes level but only the size was related to the number of KRAS copies; (6) exosomal KRAS mutated copies and fractional abundance were significantly higher in patients with liver metastases; (7) the median cut-off values of 125 of KRAS wild-type copies/ml and 37 for KRAS mutated-ones were identified as useful prognostic factors related to OS. Overall, these data showed the relationship among plasma exosomes levels and their genetic content with some clinical features of mCRC patients.

In our study we did not isolate tumor-derived exosomes but analysed all circulating exosomes. Several studies have demonstrated that it is not necessary to do so since most of the exosomes present in body fluids in cancer patients are tumor-derived^23^. We showed that the concentration of total exosomes could have relevance for the management of metastatic colorectal cancer patients potentially with approach/procedures not so much complicated and easily available in clinical cancer laboratories. So far specific biomarkers of tumor-derived exosomes have not yet been discovered, even if some proteins are more enriched in specific tumor exosomes (i.e., CD147 on exosomes released from colorectal cancer cells)^26^. Surely, the absence of specific biomarkers could represent a limitation of our study. Moreover, we hypothesize that tumor spread to other body organs increases the amount of exosomes released in the circulation and showed that the concentration in the metastatic patients is higher compare to localized primary tumor. In any case, the limitation of our study is the sample size of primary localized tumors (29) versus metastatic cases (70) so results must be taken with caution and further studies are needed to confirm them.

We showed that in mutated patients there is an increased amount of circulating exosomes at progression. We explained this result with the increased tumor burden. The most striking, significant increase, in mutated patients might be related to a more marked progression (i.e., an increased tumor burden) in mutated patients or by an increased ability of the therapy used for mutated patients (bevacizumab) to induce a rebound reaction stimulating exosomes release. Moreover, the higher exosomes levels in patients with multiple lesions and bigger tumors likely correlate with the increased tumor burden. Despite these results further studies will be needed to solve this question.

Ongoing trials are evaluating the disappearing of KRAS mutated clone after antiangiogenic chemotherapy and the possibility top use anti-EGFR treatment after a first-line progression also in initially RAS mutant patients^20^. Recently, Klein-Scory and colleagues showed that the KRAS mutated clones, assessed by ctDNA, disappeared after chemotherapy^17^. We confirmed this finding adopting exosome-based liquid biopsy, demonstrating that RAS mutations rapidly disappeared in patients after the first cycles of chemotherapy (post-therapy), although they were detectable again in 80% of cases at progression.

The emergence of RAS-mutant clones in the plasma of patients with initially wilde-type RAS tumors has been also widely described, inducing a close monitoring of the onset of secondary resistance to anti-EGFR therapy and generating new hypotheses for blood- guided therapeutic strategies^16,27^ We identified 12% of cases in which patients tested RAS WT by tissue biopsy of the primary tumor were found to be mutated in metastatic disease as assessed by exosome-based liquid biopsy. The exciting questions are whether: (1) patients with RAS-mutated tumors at diagnosis but with disappearance of RAS mutations in blood during therapy would benefit from treatment with anti-EGFR monoclonal antibodies analogously to RAS wild-type tumors at diagnosis; (2) a close monitoring of the onset of secondary resistance to anti-EGFR therapy is warranted to stop earlier this treatment to avoid: overtreatment, waste money for health care systems and more time to study what kind of secondary resistance has developed and how to manage it clinically.

**Figure 4:**
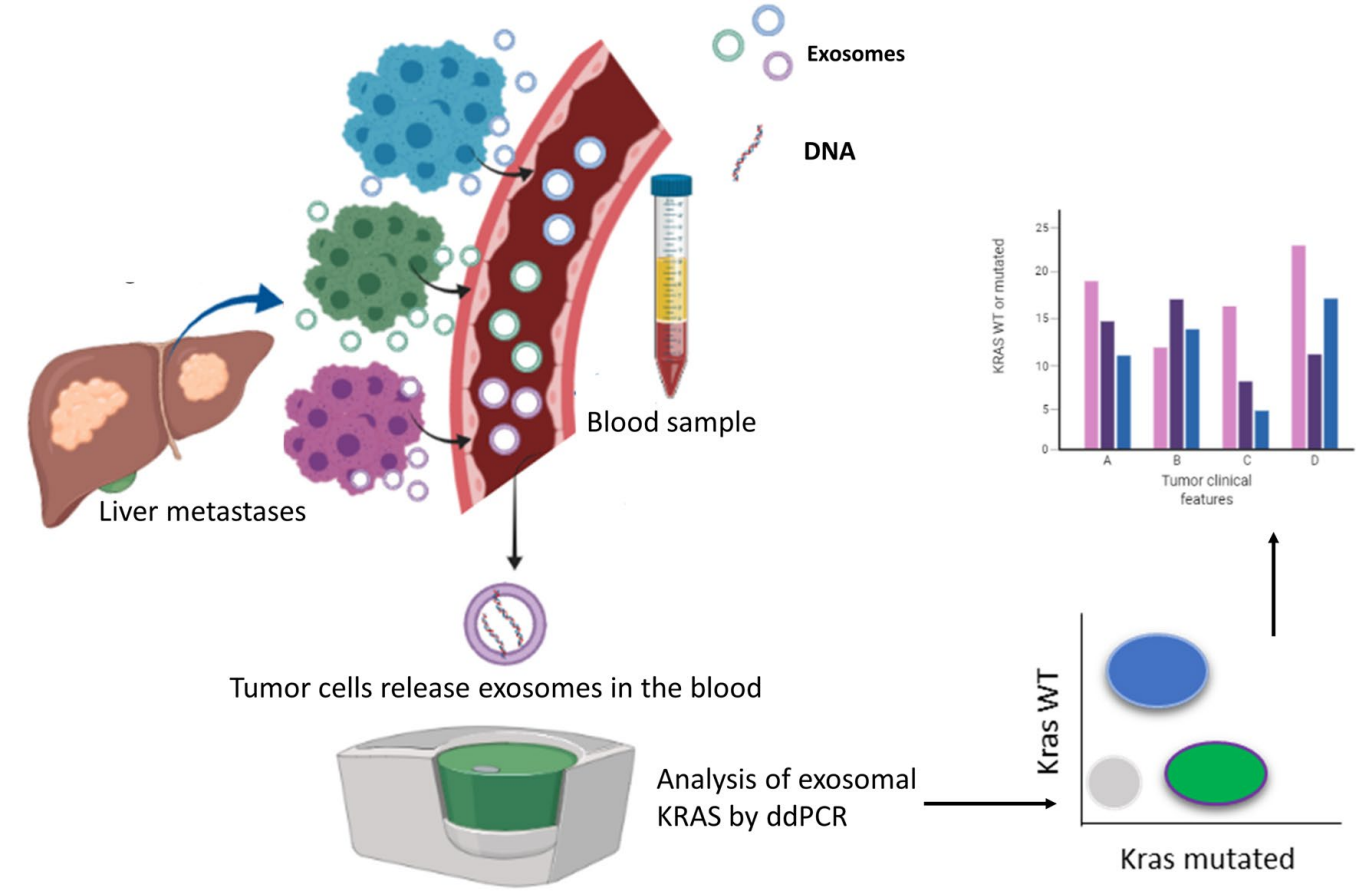
Scheme of exosome-based liquid biopsy (created with BioRender.com).

Our prospective study showed that plasma exosomal DNA might be used as good and innovative tool to monitor the mutational status of mCRC patients during the treatment and to predict disease prognosis. Metastatic cancer cells release in the blood exosomes that carry DNA with the same mutational profile of producing cancer cells. The isolation of exosomes from blood sample allowed us to analyze by ddPCR the mutational status of exosomal KRAS and to identify the relationship among plasma exosomes levels and their genetic content with some clinical features of mCRC patients that could be relevant to therapeutic stratification (Fig. 4).

Limitations of our study are: (1) the high standard error in statistical analyses likely related to the relatively small sample size of our cohort; (2) it is not possible to establish with certainty if the DNA is not detectable or if really a complete nucleotide exchange has occurred but probably the first option is the most accredited one. So, the absence of detectable RAS mutations in plasma cannot certainly exclude that a RAS mutation might be present in the sample below the assay limit of detection. Nevertheless, we believe that this is not noteworthy from a clinical point of view since it has been showed, by studies on cfDNA, that patients with a low RAS mutant fraction might still benefit from the addition of cetuximab to chemotherapy^5^; (3) assessing protein amount for exosomes quantification but several studies demonstrated its reliability^23^.

To our knowledge, this is the first study analyzing KRAS mutational status using plasma exosomes in mCRC, but its significance is limited by the low number of patients enrolled. A study with a larger cohort of mCRC patients is warranted to confirm these preliminary interesting data.

## Supplementary Information

Supplementary Figures legends.

Supplementary Figure 1.

Supplementary Figure 2.

Supplementary Figure 3.

Supplementary Figure 4.

Supplementary Figure 5.

Supplementary Table 1.

Supplementary Materials and Methods.

## Data Availability

All data relevant to the study are included in the paper.

## Abbreviations

mCRC: Metastatic colorectal cancer
OS: Overall survival
CRC: Colorectal cancer
anti-EGFR: EGFR-targeted antibodies
cfDNA: Cell free DNA
exoDNA: Exosomal DNA
post-4: After 4 days from surgery
post-30: After 30 days from surgery
basal sample: Before first chemotherapeutic administration
post-therapy: At each following radiological evaluation
progression: At radiological progression
ddPCR: Droplet digital PCR
FA: Fractional abundance
SD: Stable disease
RP: Responded partially
CSC: Colorectal cancer stem cell lines
