# Supplementary material for: Mutational status of plasma exosomal KRAS predicts outcome in patients with metastatic colorectal cancer
Source: Sci Rep. 2021 Nov 22;11:22686. doi: 10.1038/s41598-021-01668-7 (PMC8608842; doi:10.1038/s41598-021-01668-7)
Supplement: Supplementary file 1 — Supplementary Figures legends. [file 41598_2021_1668_MOESM1_ESM.docx]

**Supplementary figures legend**

**Supp. Fig. 1**. a) DLS of exosomes isolated by ultracentrifugation (red round circle) and Invitrogen commercially avaiable kit (green round circle); b) morphological comparison by TEM of exosomes extracted by ultracentrifugation (ultra) and Invitrogen (Inv) commercially avaiable kit; c) western blot analysis of exosomal marker CD9, CD63 and non-exosomal marker β-actin in exosomes obtained by ultracenrifugation protocol (ultra) and Invitrogen kit (Inv).

**Supp. Fig. 2** Size evaluation of exosomal DNA isolated by QIAamp or virus kit (a-b); goodness of DNA tested by enzyme digestion (c-d-e).

**Supp. Fig. 3** Study overview and patient stratification.

**Supp. Fig. 4** Evaluation of KRAS copies wild-type and mutated (*G12D/V* mutation, respectively in CSC3 and CSC2; KRAS WT in CSC1 cells) to confirm the correct KRAS gene detection. CSC= colorectal cancer stem cells.

**Supp. Fig. 5** Expression of KRAS WT and mutated clones at basal and at progression.
