# Supplementary figures and images for: Mutational status of plasma exosomal KRAS predicts outcome in patients with metastatic colorectal cancer
Source: Sci Rep. 2021 Nov 22;11:22686. doi: 10.1038/s41598-021-01668-7 (PMC8608842; doi:10.1038/s41598-021-01668-7)

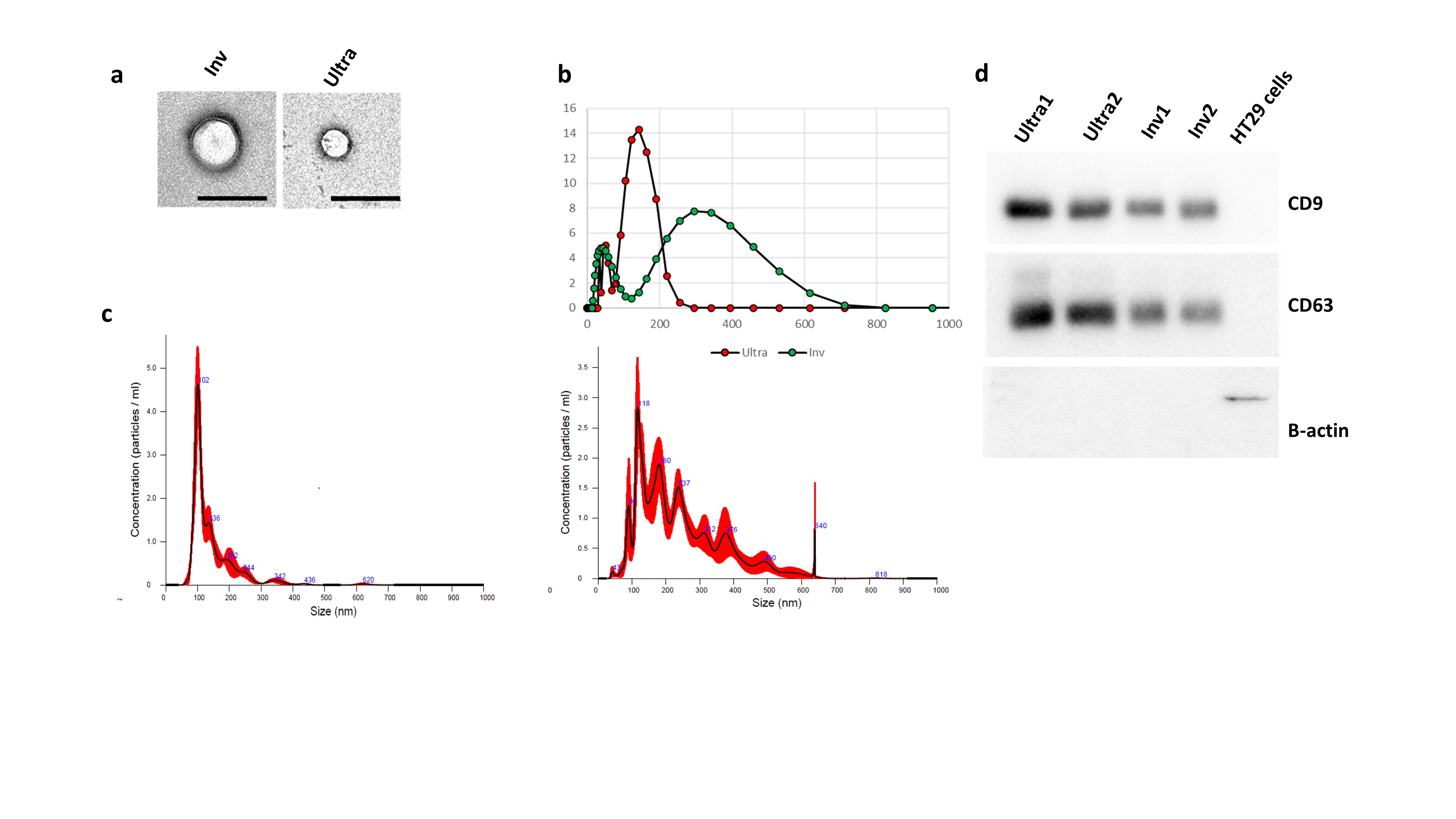

Supplement: Supplementary file 2 — Supplementary Figure 1. [file 41598_2021_1668_MOESM2_ESM.tif]

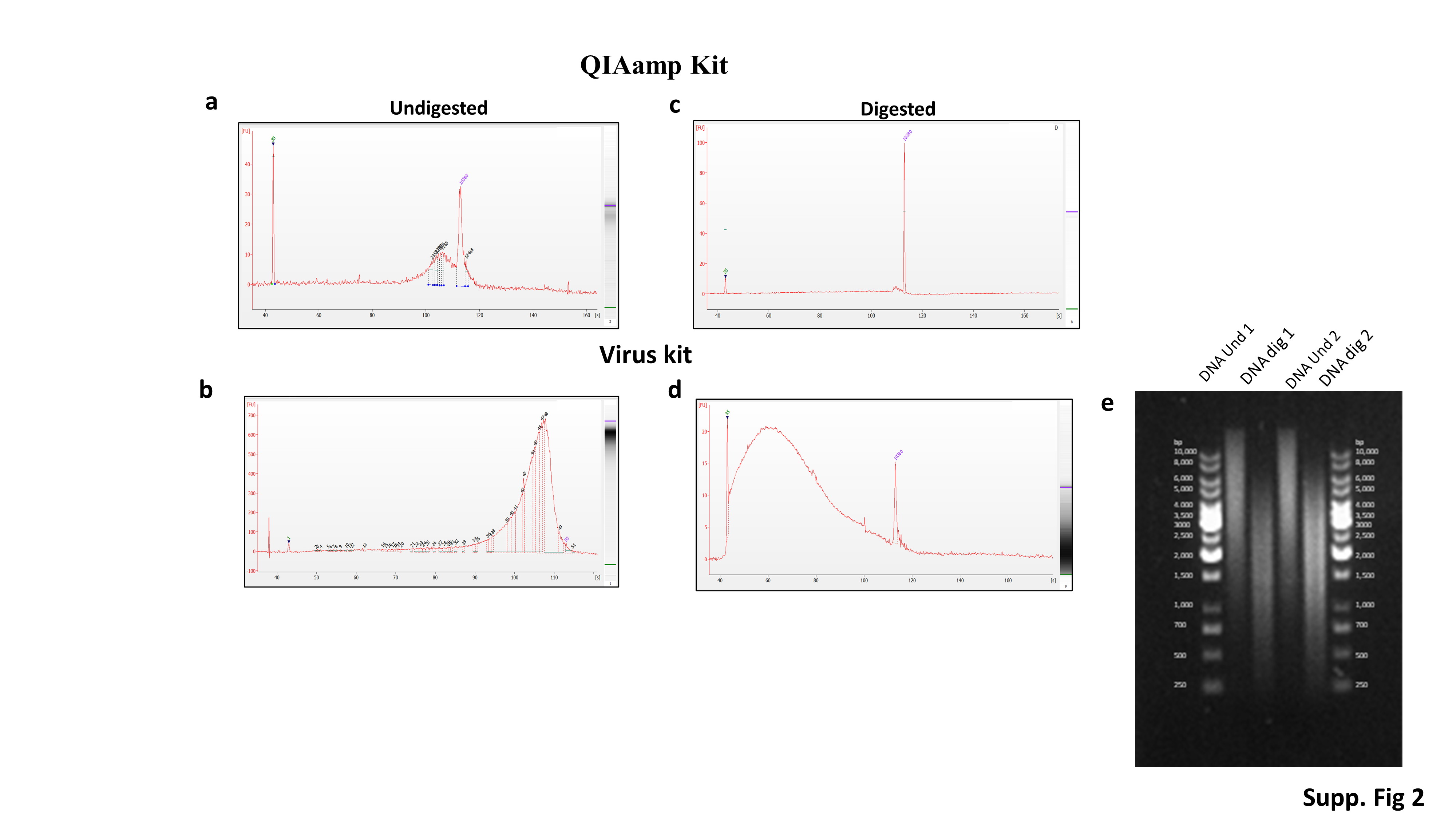

Supplement: Supplementary file 3 — Supplementary Figure 2. [file 41598_2021_1668_MOESM3_ESM.tif]

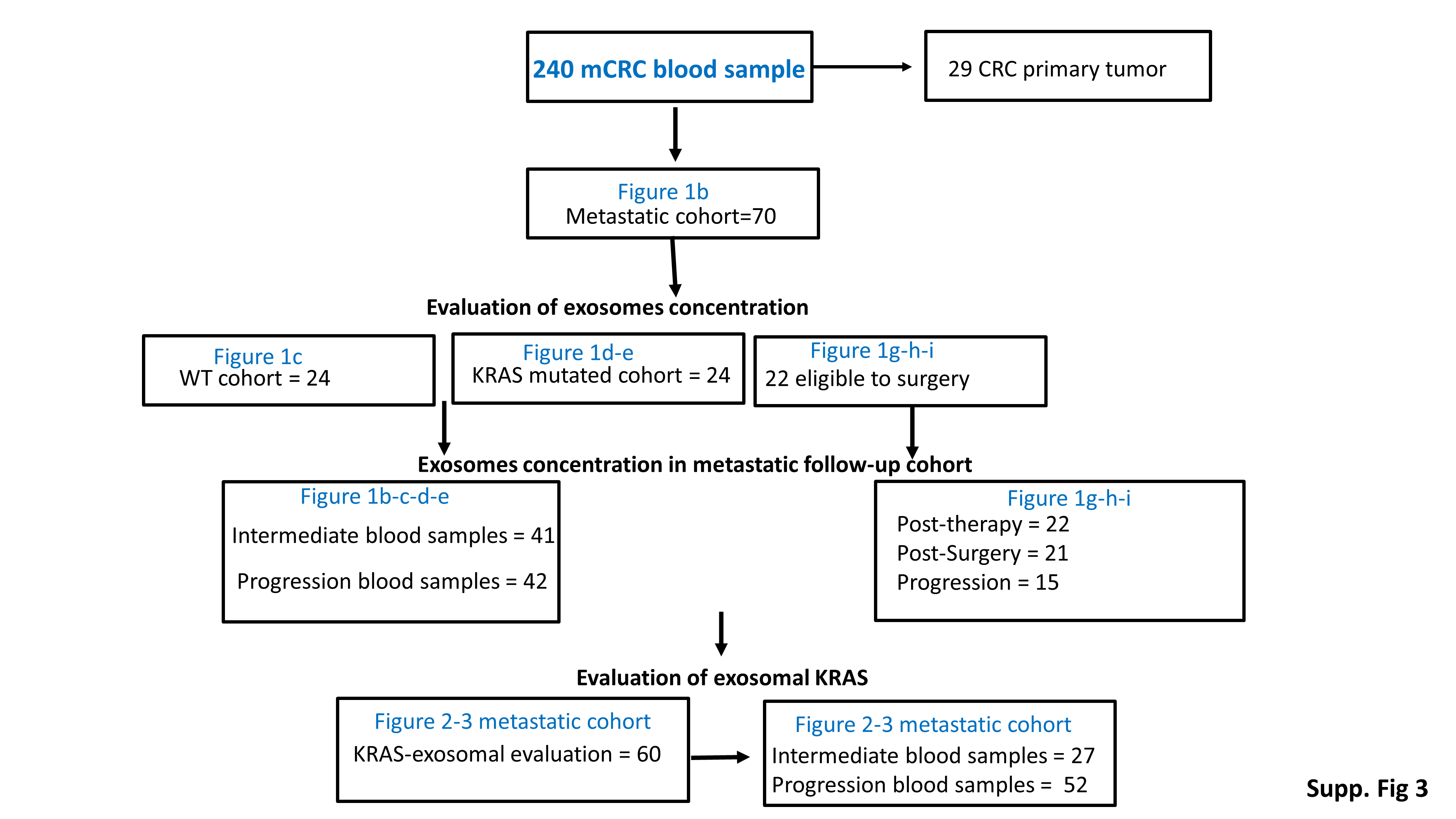

Supplement: Supplementary file 4 — Supplementary Figure 3. [file 41598_2021_1668_MOESM4_ESM.tif]

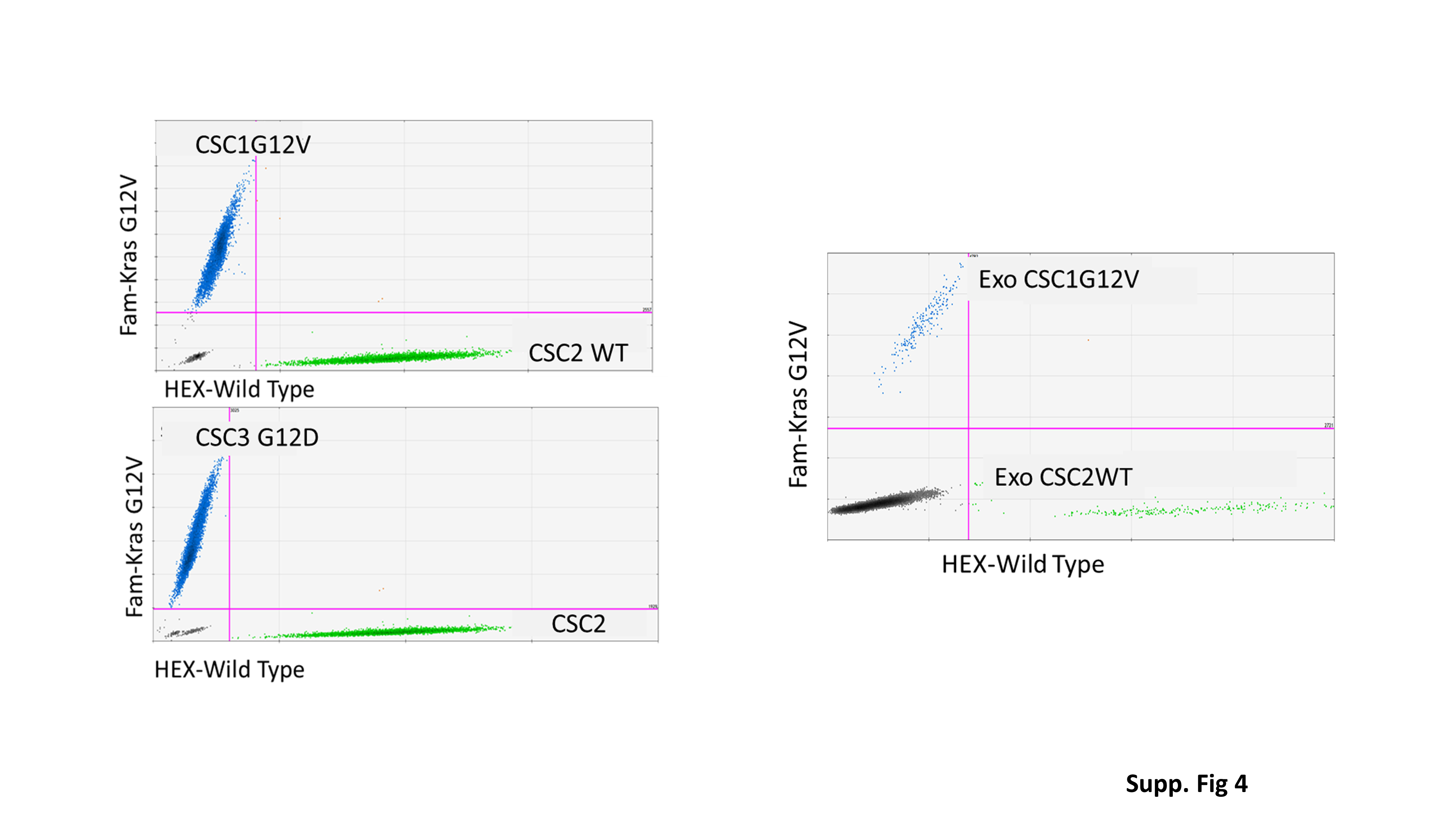

Supplement: Supplementary file 5 — Supplementary Figure 4. [file 41598_2021_1668_MOESM5_ESM.tif]

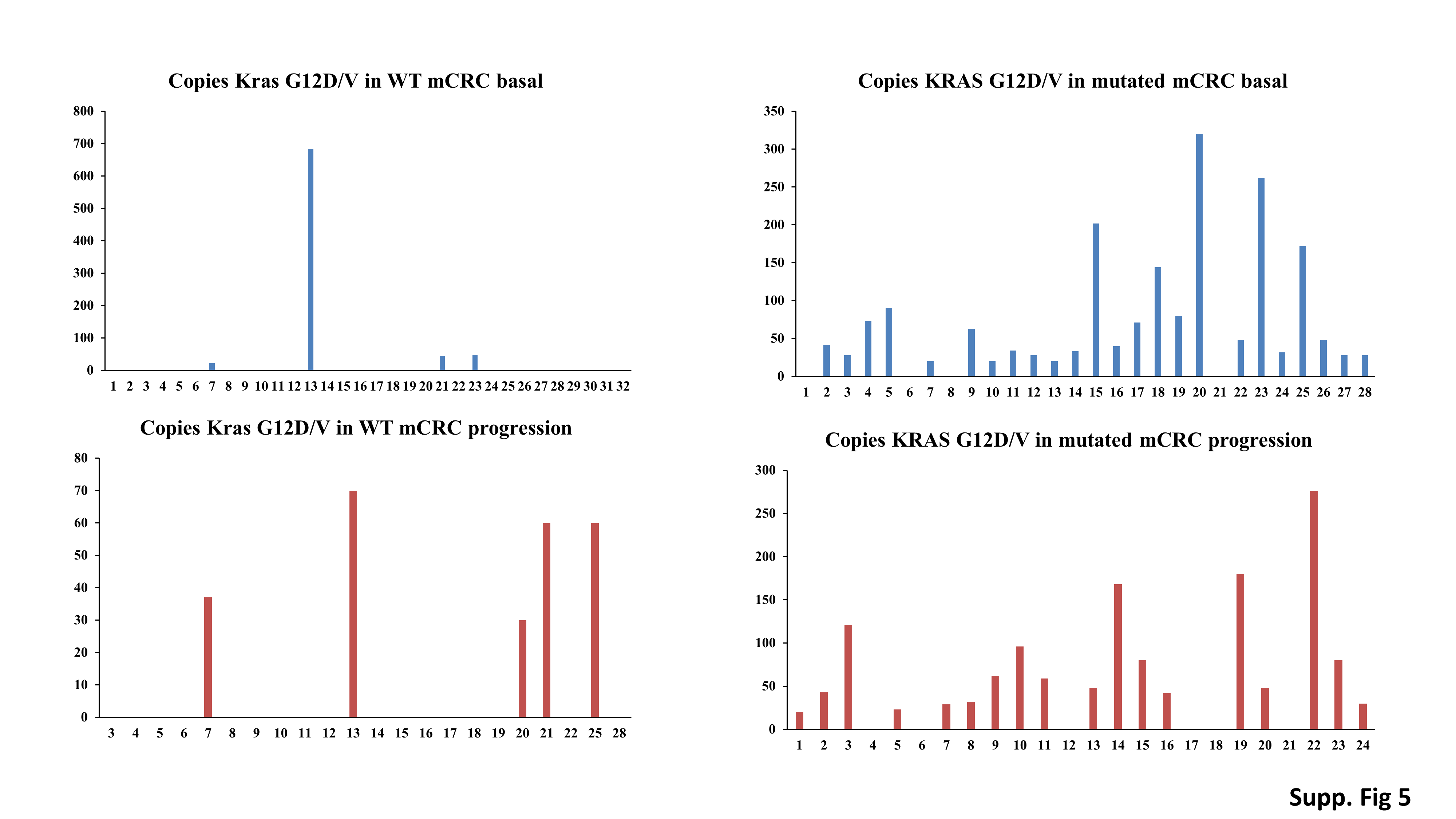

Supplement: Supplementary file 6 — Supplementary Figure 5. [file 41598_2021_1668_MOESM6_ESM.tif]
