# Supplementary material for: Mutational status of plasma exosomal KRAS predicts outcome in patients with metastatic colorectal cancer
Source: Sci Rep. 2021 Nov 22;11:22686. doi: 10.1038/s41598-021-01668-7 (PMC8608842; doi:10.1038/s41598-021-01668-7)
Supplement: Supplementary file 7 — Supplementary Table 1. [file 41598_2021_1668_MOESM7_ESM.docx]

| Enrolled patients | N=70 |
| --- | --- |
| Localization of primary tumor   - colon left - colon right - rectum | 42  23  5 |
| Number of lesions  <3  >3 | 19  51 |
| Liver metastases | 56 |
| Lung metastases | 13 |
| Peritoneum metastases | 15 |
| Primary tumor resection   - yes - no | 44  26 |
| RAS in primary tumor   - Wild type - KRASG12D/V | 37  33 |
| CEA   - Positive (>5ng/ml) - Negative (<5ng/ml) | 39  31 |

**Supp. Table 1. Clinical characteristics of patients**
