# Supplementary material for: Mutational status of plasma exosomal KRAS predicts outcome in patients with metastatic colorectal cancer
Source: Sci Rep. 2021 Nov 22;11:22686. doi: 10.1038/s41598-021-01668-7 (PMC8608842; doi:10.1038/s41598-021-01668-7)
Supplement: Supplementary file 8 — Supplementary Materials and Methods. [file 41598_2021_1668_MOESM8_ESM.docx]

**Supplementary Material and Methods**

**Western blot analysis**

Exosomal proteins were extracted by lysing of 10 µl of exosomes with 10 µl NP40 0,25% for 60’ in ice, vortexing six time. Exosome concentration was estimated by Bradford assay. About 10 μg of protein from each sample was separated under non-reducing conditions on a 10 % SDS-PAGE gel, transferred to a polyvinylidene fluoride (PVDF) membrane, and blotted with an antibody against CD9 (dilution, 1:500; Santa Cruz Biotechnology, Inc., Dallas, TX, USA).

**Extraction of DNA from cell-free plasma and exosomes**

QIAamp1Circulating Nucleic Acid Kit (Qiagen, Santa Clarita, CA) and QIAamp MinElute Virus Spin Kit (Qiagen, Santa Clarita, CA) were used for the isolation of exosomal DNA isolated from 2ml of plasma and suspended in 200µl of PBS (0.1 µm filtered). The manufacturer's recommended protocol was followed to isolate exosomal DNA from 100µl of started exosomes sample diluted in 1XPBS (0.1 µm filtered). DNA was eluted in 50 μL of elution buffer and stored at -80°C until use.

**Analysis of exosomal DNA by Agilent Bioanalyzer**

Exosomal DNA was extracted from blood as described above. DNA extracted from exosomes was treated with RNase as described above and analyzed using Agilent Bioanalyzer 2100 instrument and Agilent High Sensitivity DNA Kit following manufacturer's recommended protocol. The Agilent 2100 Expert software analyses DNA profile of each sample automatically and displays electropherogram for each sample.

**Agarose gel separation of exosomal DNA**

Exosomal DNA was extracted as described. Eluted DNA was divided into two aliquots, one was treated with cocktail of digestion enzyme and the other aliquot was not treated. Treated and untreated DNA was separated using 1% agarose gel, stained with SYBR™ Safe DNA Gel Stain (Thermo-Fisher Scientific), and visualized using Bio-Rad ChemiDoc™ XRS+ system.

**Total DNA quantification**

DeNovix dsDNA High Sensitivity Assay was used to quantify DNA isolated from plasma exosomes by QFX Fluorometer (DeNovix) following manufacturer's recommended protocol.

**Nanoparticle Tracking analysis (NTA)**

Size and concentration of EVs were defined using NanoSight NS300 (Malvern Panalytical Ltd, UK). Samples were diluted 1:10 in PBS in a final volume of 500 µL. Data were processed using NTA 3.2 software (Malvern Panalytical Ltd).
